# Device-Embedded Cameras for Eye Tracking–Based Cognitive Assessment: Validation With Paper-Pencil and Computerized Cognitive Composites

**DOI:** 10.2196/11143

**Published:** 2018-07-24

**Authors:** Nicholas Bott, Erica N Madero, Jordan Glenn, Alexander Lange, John Anderson, Doug Newton, Adam Brennan, Elizabeth A Buffalo, Dorene Rentz, Stuart Zola

**Affiliations:** ^1^ Clinical Excellence Research Center Department of Medicine Stanford University School of Medicine Stanford, CA United States; ^2^ Neurotrack Technologies, Inc Redwood City, CA United States; ^3^ Department of Physiology and Biophysics University of Washington Seattle, WA United States; ^4^ Department of Neurology Massachusetts General Hospital Boston, MA United States; ^5^ Department of Neurology Brigham and Women's Hospital Boston, MA United States; ^6^ Office of the Provost Emory University Atlanta, GA United States

**Keywords:** eye tracking, visual paired comparison, preclinical Alzheimer’s disease, neuropsychological testing

## Abstract

**Background:**

As eye tracking-based assessment of cognition becomes more widely used in older adults, particularly those at risk for dementia, reliable and scalable methods to collect high-quality data are required. Eye tracking-based cognitive tests that utilize device-embedded cameras have the potential to reach large numbers of people as a screening tool for preclinical cognitive decline. However, to fully validate this approach, more empirical evidence about the comparability of eyetracking-based paradigms to existing cognitive batteries is needed.

**Objective:**

Using a population of clinically normal older adults, we examined the relationship between a 30-minute Visual Paired Comparison (VPC) recognition memory task and cognitive composite indices sensitive to a subtle decline in domains associated with Alzheimer disease. Additionally, the scoring accuracy between software used with a commercial grade eye tracking camera at 60 frames per second (FPS) and a manually scored procedure used with a laptop-embedded web camera (3 FPS) on the VPC task was compared, as well as the relationship between VPC task performance and domain-specific cognitive function.

**Methods:**

A group of 49 clinically normal older adults completed a 30-min VPC recognition memory task with simultaneous recording of eye movements by a commercial-grade eye-tracking camera and a laptop-embedded camera. Relationships between webcam VPC performance and the Preclinical Alzheimer Cognitive Composite (PACC) and National Institutes of Health Toolbox Cognitive Battery (NIHTB-CB) were examined. Inter-rater reliability for manually scored tests was analyzed using Krippendorff’s kappa formula, and we used Spearman’s Rho correlations to investigate the relationship between VPC performance scores with both cameras. We also examined the relationship between VPC performance with the device-embedded camera and domain-specific cognitive performance.

**Results:**

Modest relationships were seen between mean VPC novelty preference and the PACC (r=.39, *P*=.007) and NIHTB-CB (r=.35, *P*=.03) composite scores, and additional individual neurocognitive task scores including letter fluency (r=.33, *P*=.02), category fluency (r=.36, *P*=.01), and Trail Making Test A (–.40, *P*=.006). Robust relationships were observed between the 60 FPS eye tracker and 3 FPS webcam on both trial-level VPC novelty preference (r=.82, *P*<.001) and overall mean VPC novelty preference (r=.92 *P*<.001). Inter-rater agreement of manually scored web camera data was high (kappa=.84).

**Conclusions:**

In a sample of clinically normal older adults, performance on a 30-minute VPC task correlated modestly with computerized and paper-pencil based cognitive composites that serve as preclinical Alzheimer disease cognitive indices. The strength of these relationships did not differ between camera devices. We suggest that using a device-embedded camera is a reliable and valid way to assess performance on VPC tasks accurately and that these tasks correlate with existing cognitive composites.

## Introduction

Alzheimer disease (AD) and other forms of dementia, broadly characterized by declines in mental ability severe enough to interfere with daily life, pose serious challenges to patients, caregivers, and healthcare systems worldwide. As populations age, the global prevalence of dementia is expected to triple to 132 million between 2015 and 2050 [1]. In the United States (US) alone, the costs of AD are projected to grow by 400% from US $186 billion in 2018 to US $750 billion in 2050 as the number of people with dementia increases from 5.5 million to 13.8 million [2]. Alzheimer disease can go undetected for long periods of time because the disease has a prolonged preclinical phase, during which neuronal and neurobiological changes can occur for years or decades before noticeable symptoms appear. Early detection of AD during the preclinical phase has the potential to decrease medical and long-term care costs by as much as US $7 trillion in the US [2]. Detection of preclinical AD can enable people to seek treatment earlier, address modifiable risk factors, and potentially slow the progression of the disease, ultimately preserving cognitive function and reducing population health care costs [1,2].

Current detection methods for preclinical AD include the use of biomarkers, such as neuroimaging and cerebrospinal fluid tests for amyloid-β and tau proteins [3,4]. Increasingly, cognitive assessment composites targeting relevant cognitive domains sensitive to AD pathologies, such as the Preclinical Alzheimer’s Cognitive Composite (PACC) [5] and National Institutes of Health Toolbox Cognitive Battery (NIHTB-CB) [6,7], have shown efficacy in stratifying preclinical AD populations. However, there are drawbacks to both detection methods that ultimately limit their feasibility for screening large populations. For example, biomarker tests are expensive and invasive, cognitive batteries require trained staff for standardized administration, and both methods restrict access by requiring people to travel to a clinic.

Another method for detecting presymptomatic cognitive decline is through the use of eye tracking systems that assess eye movement behavior [8]. For example, eye tracking–based tasks that assess saccade patterns can be used to detect mild cognitive impairment (MCI) and AD [9,10]. Saccades, or small rapid movement of the eyes between fixations of relevant stimuli, can be examined within certain task paradigms to quantify inhibitory control. Another eye tracking-based task of particular interest is the visual paired-comparison (VPC) task, which has been shown to reliably detect early signs of cognitive decline in older adults before symptoms are present [11,12]. Visual paired-comparison tasks are a well-established method for detecting memory dysfunction in humans and other primates, from infancy through adulthood [11-18].

Commercial-grade eye-tracking cameras have traditionally been used to collect VPC data. These high frame rate cameras can capture a variety of complex visual features, including saccades, smooth pursuit (consistent tracking movements of the eyes), and fixation distributions (eyes focusing on particular areas or items), which can all be assessed for abnormal patterns indicative of a variety of pathologies [19,20]. Data can either be analyzed automatically with software provided by the manufacturer or inspected manually by researchers who have experience evaluating eye-tracking metrics. However, the use of commercial eye trackers as a cognitive health screening tool has limitations, much like biomarker tests and traditional cognitive assessments. Eye-tracking devices are expensive, complicated to use, and are not widely available in clinical settings. To effectively reach the growing number of individuals at risk for cognitive decline, preclinical AD screening tests that are both reliable and scalable, such as VPC tests that utilize ubiquitous device-embedded webcams (eg, mobile phones, tablets, laptops), need to be validated and implemented.

Emerging research indicates that when used for cognitive tests sensitive to early signs of decline, embedded webcams in laptops and mobile devices produce data of similar quality to that collected by commercial-grade cameras [21]. It remains to be determined if scoring accuracy will be maintained for a longer (30 minutes) version of the test. The 30-minute VPC task has been shown to reliably predict future declines in cognitive status among clinically normal individuals and individuals with amnestic MCI, a subtype of MCI with focal deficits in learning and memory performance [12]. The validation of accurate scoring methods for the embedded camera version of the test and its relationship to paper-pencil and computerized cognitive composites would add considerable value to the task as an asset to a clinician’s assessment repertoire.

The purpose of this study is (1) to investigate the relationships between performance on a 30-minute webcam-based digital VPC task and two cognitive composite indices sensitive to subtle impairment in AD-relevant cognitive domains, (2) to examine the relationship between performance on the VPC task with a device-embedded camera and domain-specific cognitive scores, and (3) to investigate the accuracy of human-coded gaze positions on a thirty-minute VPC using a laptop-embedded camera when compared to an automatically scored gold standard high frame rate eye-tracking camera.

## Methods

### Participants and Procedures

All subjects underwent informed consent procedures approved by the Partners Human Research Committee, the Institutional Review Board for Brigham and Women’s Hospital and Massachusetts General Hospital. A total of 49 clinically normal, community-dwelling older adults were recruited from a cohort of volunteers interested in participating in research studies at the Center for Alzheimer Research and Treatment at Brigham and Women’s Hospital and the Massachusetts Alzheimer Disease Research Center at Massachusetts General Hospital. Subjects were excluded if they had a history of alcoholism, drug abuse, head trauma, or current serious medical or psychiatric illnesses. All subjects above the age of 50 years and within age-specified norms on the Telephone Interview of Cognitive Status [22] were eligible for the study. No prior computer or iPad knowledge was required to participate. Subjects attended 1 clinic visit, during which they completed paper-pencil based cognitive tasks including the PACC, the NIHTB-CB, and the Neurotrack 30-minute VPC eye-tracking assessment. Eye-tracking data for the 30-minute VPC task was collected simultaneously by a commercial-grade eye tracker and a laptop-embedded camera.

### Cognitive Composites

The PACC is a paper-pencil cognitive composite that includes 2 tasks of episodic memory, a task of speeded executive functioning, and a global cognitive screen. The Logical Memory-delayed recall score and the Free and Cued Selective Reminding Test total score comprised the episodic memory tests, with the Wechsler Adult Intelligence Scale-Revised Digit Symbol Coding Test total score representing the speeded executive functioning measure, and the Mini-Mental State Exam total score serving as the global cognitive screen [23].

Additionally, a measure of attention and processing speed (Trail Making Test A) and 2 measures of executive functioning (letter and verbal fluency) were administered. All tests were z-transformed using the performance means and standard deviations of clinically normal older adults (n=256, age range: 61-90 years) [24,25]. All four z-transformed variables were averaged together to produce a PACC composite score, with higher scores indicating better performance.

The NIHTB-CB is a computerized cognitive composite comprised of the Picture Vocabulary Test (PVT), the Flanker Inhibitory Control and Attention Test (Flanker), the Dimensional Change Card Sort Test (DCCS), the Pattern Comparison Processing Speed Test (PCPST), and the Picture Sequence Memory Test (PSMT) [7]. The PVT is a measure of receptive vocabulary, requiring participants to select from 4 images the 1 closest to the meaning of an orally presented word. The Flanker is a measure of cognitive control, requiring participants to focus on a stimulus surrounded by 4 identical stimuli around the target and having them select the direction in which the target stimulus is pointing. The DCCS is a measure of executive control, requiring participants to shift set matching a target visual stimulus to stimuli by shape or color. The PCPST is a measure of processing speed, requiring participants to rapidly match an object by shape or color. The PSMT is a measure of visual episodic memory, requiring participants to re-create the order of a set of images over 2 test trials [7]. The Flanker, PCPST, DCCS, and PSMT were scored per NIHTB-CB guidelines, and overall performance was quantified by a theta score, calculated by combining all of the scores on the individual tasks.

### Visual Paired-Comparison Test Construction

A 30-minute VPC task developed by Neurotrack Technologies Inc (Redwood City, CA) was used in this study. VPC tasks quantify how the test subject splits attention between familiar and novel visual stimuli, with a familiarization phase preceding a testing phase. During the familiarization phase, subjects were presented with pairs of identical visual stimuli for a fixed period (5 seconds). During the test phase, which follows a delay of either 2 seconds or 2 minutes to assess immediate and delayed recognition memory, subjects were presented with additional pairs of visual stimuli, including 1 from the familiarization phase (familiar stimulus) and 1 novel stimulus. The ratio of time a subject spends gazing at the novel stimulus relative to the total viewing time produces a novelty preference (NP) score, with higher scores representing better declarative memory function and lower scores indicating impaired function [11,26,27].

### System Components

Eye movements during the VPC task were simultaneously recorded with a commercial-grade Tobii X2-60 eye tracker camera system (Tobii AB, Stockholm, Sweden) and an embedded web camera on a 13-inch Apple MacBook Air laptop (Apple, Cupertino, CA). The Tobii camera sampled at 60 Hz, with corneal and pupil centers determining the gaze angle. Eye data were recorded using the Tobii SDK and API software. Participants were seated approximately 27 inches from the 13-inch laptop monitor that displayed the visual stimuli. The Apple MacBook Air laptop processor was a 1.4 GHz Intel Core i5 with 4 GB 1,600 MHz DDR3 memory and a 1,536 MB Intel HD Graphics 5,000 Graphics card. Video resolution of the laptop during test recording was 640 by 480.

### Calibration Validation and Gaze Position

Explanations regarding the validation of camera calibration, data acquisition, and fixation filters for device-embedded cameras have previously been reported [21]. Briefly, before the start of the VPC task, subjects were instructed to watch a blue dot travel around the screen. Acting as a coordinate system, the top left of the screen represented (0, 0) and the bottom right of the screen represented (1, 1). The calibration ball traveled a predetermined path: (0.5, 0.5), (0.1, 0.1), (0.1, 0.9), (0.9, 0.9), (0.5, 0.5), (0.9, 0.1), (0.5, 0.1), (0.1, 0.5), (0.5, 0.9), pausing at each of the above points for approximately 2 seconds. Calibration validation of the device-embedded camera was determined by three human coders evaluating the individual frames of the calibration-phase video. Coding of the calibration phase video was repeated if individual accuracy of correctly coded calibration frames was below 90%. Calibration data were used to generate individualized models to predict gaze location and duration but were not incorporated into the experimental procedure. Calibration validation of the commercial grade eye-tracking camera was determined by multiple accuracy metrics produced by the Tobii X2-60 SDK/API software.

Using the Tobii Pro Analytics software development kit default Tobii Pro Studio settings, we utilized the Active Display Coordinate System and the User Coordinate System to determine gaze location. Each data point consisted of an estimated gaze point for both the left and right eye. For each data point, the midpoint of the 2 gaze points was used as the definitive gaze estimate. Test trials were automatically excluded if more than 4s of data was missing due to the Tobii failing to find the eyes of the subject. In a previous study, Zola and colleagues used an Applied Science Laboratories eye tracker that recorded gaze data at 120 Hz (120 frames per second) [12]. To replicate the cluster-based algorithm used by Zola et al [12], a fixation filter to process the raw Tobii data was developed [21]. Three researchers with expertise in eye-tracking behavior and the Tobii X2-60 eye tracker system independently inspected all test trials to ensure the quality of test data. Test trials flagged for aberrant gaze paths (eg, gaze clustering, erratic saccades) were discussed corporately, and a consensus decision was made to retain or discard the trial in question.

In addition to the commercial eye tracker, subjects were simultaneously recorded with a device embedded camera during the calibration and test phases. A high definition Flash video recorder recorded the subject, and the resulting Flash video (FLV) footage was streamed to Neurotrack’s Wowza Amazon Web Services instance. Metadata, such as calibration phase timing and timing of task image presentation, was injected into the FLV video to ensure correspondence between frames of the video and events of the test.

### Scoring

Performance on preferential looking VPC tasks is quantified as novelty preference. In the present study, novelty preference was defined as the percentage of time the participant spent looking at the novel image compared with the familiar image. For each test trial, NP was calculated as (time viewing novel image) divided by (total time viewing either image). Mean novelty preference for each of the 20 test trials yielded the overall novelty preference score. Using the commercial grade eye-tracking camera, a rectangular area of interest perimeter slightly larger than each image was defined. Gaze time on each image was calculated based on the total gaze fixation time recorded by the Tobii X2-60 software.

For the device-embedded camera data, individual processed video frames were evaluated on a frame by frame basis down-sampled to 3 frames per second (FPS) by 3 independent human coders to determine whether the subject was looking to the left, right, or neither side of the screen. Coding of the “neither” option was intended for frames when the participant was blinking, or when the image was of poor enough quality that the iris was indistinguishable from the rest of the eye. For each image, the majority decision was taken by the individual ratings. The NP score for each trial was the percentage of frames that the participant was rated as looking at the novel side (no. of “novel” frames) divided by (total no. of “novel” frames + no. of “familiar” frames).

### Visual Paired-Comparison Data Analysis

Analyses of VPC test data were conducted with IBM SPSS version 24.0 using non-parametric statistical procedures due to the non-normal distribution of VPC test performance. Inter-rater agreement of web camera data scoring was assessed using Krippendorff’s kappa calculation [28]. Relationships between the Tobii X2-60 eye-tracking camera (60 FPS) and the laptop embedded camera (3 FPS) were assessed using Spearman’s Rho correlations. Relationships between VPC task performance, paper-pencil based neuropsychological tasks and computerized neuropsychological tasks were assessed using two-tailed Spearman’s Rho correlations. There were no relationships between age, gender or education on the VPC task or individual paper-pencil based neuropsychological tasks. Performance on computerized neuropsychological tasks on the NIHTB-CB was assessed based on standardized scores. The Cohen standard was used to determine the strength of these relationships with correlation coefficients of .10 as weak, .30 as moderate, and .50 and above as strong [29]. The strength of inter-rater reliability kappa statistic was determined with reliability of .40 to .59 as weak, .60 to .79 as moderate, and .80 to .90 as strong.

## Results

### Participant Characteristics

Subjects were all cognitively normal community-dwelling older adults. The age range of the study cohort was 54-97 years and the level of education ranged from 12-20 years. (Table 1).

**Table 1:** Characteristics of the study cohort of cognitively normal older adults.

Characteristic		Value (N=49)	
Age (years), mean (SD)		69 (8)	
**Gender, n (%)**			
	Female		28 (57)
	Male		21 (43)
Years of education, mean (SD)		16 (3)	
**Race, n (%)**			
	European-American		31 (63)
	African American		18 (37)

**Table 2:** Correlations between visual paired comparison task performance by camera type and cognitive assessments.

Cognitive assessment	Tobii X2-60	*P* value^a^	Laptop-embedded Camera	*P* value^a^	Fisher r to z transformation (*P* value)
PACC^d^ Composite	.43	.005^b^	.39	.007^b^	.42
MMSE^e^	.20	.21	.13	.40	.37
LM-DR^f^	.30	.06	.25	.09	.16
FCSRT^g^	–.05	.75	.22	.15	.11
Digit Symbol Coding	.48	.001^b^	.32	.03^c^	.18
Letter Fluency	.44	.004^b^	.33	.02^c^	.28
Category Fluency	.43	.005^b^	.36	.01^c^	.36
Trails A	–.45	.003^b^	–.40	.006^b^	.39
NIHTB-CB^h^ Composite	.32	.049^c^	.35	.03^c^	.40
PVT^i^	.28	.09	.28	.07	.50
PSMT^j^	.39	.01^c^	.33	.03^c^	.38
Flanker	–.006	.97	.22	.16	.16
DCCS^k^	.29	.07	.37	.02^c^	.35

^a^Determined by Spearman correlations and two-tailed Fisher r to z correlation comparisons.

^b^*P*<.01.

^c^*P*<.05.

^d^PACC: : Preclinical Alzheimer’s Cognitive Composite.

^e^MMSE: Mini-Mental State Exam.

^f^LM-DR: Logical Memory-delayed recall.

^g^FCSRT: Free and Cued Selective Reminding Test.

^h^NIHTB-CB: National Institute of Health Toolbox Cognitive Battery.

^i^PVT: Picture Vocabulary Test.

^j^PSMT: Picture Sequence Memory Test.

^k^DCCS: Dimensional Change Card Sort.

### Correlations Between Visual Paired-Comparison Data and Cognitive Composites

To further investigate the data from the commercial-grade eye-tracking camera and the laptop-embedded camera, we compared the strength of the correlations between the Tobii X2-60 VPC and the PACC and NIHTB-CB composites with the strength of the correlations between the laptop embedded camera and the PACC and NIHTB-CB composites. Fisher r to z transformation revealed no significant differences in the strength of correlation between each modality of data acquisition (*P*>.10; Table 2).

### Associations Between Visual Paired-Comparison Data and Cognitive Domains

Performance on the NIHTB-CB was moderately correlated with scores on the PACC (r=.51, *P*<.001), which is in line with what has been published previously [6]. We also examined the correlation between VPC task performance with device-embedded camera data and cognitive test batteries. Analyses found modest relationships between VPC task performance and the PACC (r=.39, *P*=.007) and the NIHTB-CB (r=.35, *P*=.03) across 46 subjects. Three subjects were excluded from the analysis due to insufficient data quality.

We then analyzed the correlations between VPC performance and domain-specific cognitive functions (Table 2). Significant relationships were observed on digit symbol coding (r=.32, *P*=.03), Trails A (r=–.40, *P*=.006), letter fluency (r=0.33, *P*=.02), and category fluency (r=0.36, *P*=.01). A trend relationship was seen on Logical Memory Delayed Recall (r=.25, *P*=.09). On the NIHTB-CB, significant relationships were observed on PSMT (r=.33, *P*=.03) and DCCS (r=.37, *P*=.02), with a trend relationship for PVT (r=.28, *P*=.07).

### Visual Paired-Comparison Data Scoring Correlations

**Figure 1:**
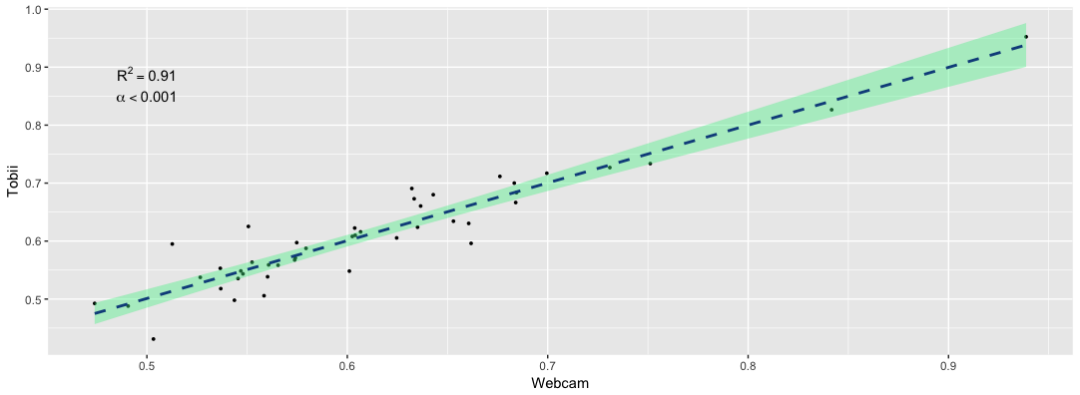
Relationship between overall mean novelty preference scores collected by the Tobii commercial-grade eye tracker and device-embedded web camera.

Analysis of the relationship between data from the commercial grade eye-tracker and the device-embedded web camera revealed strong positive associations overall. Spearman’s Rho correlation was .91 (n=44, *P*<.001) among study participants (Figure 1).

**Figure 2:**
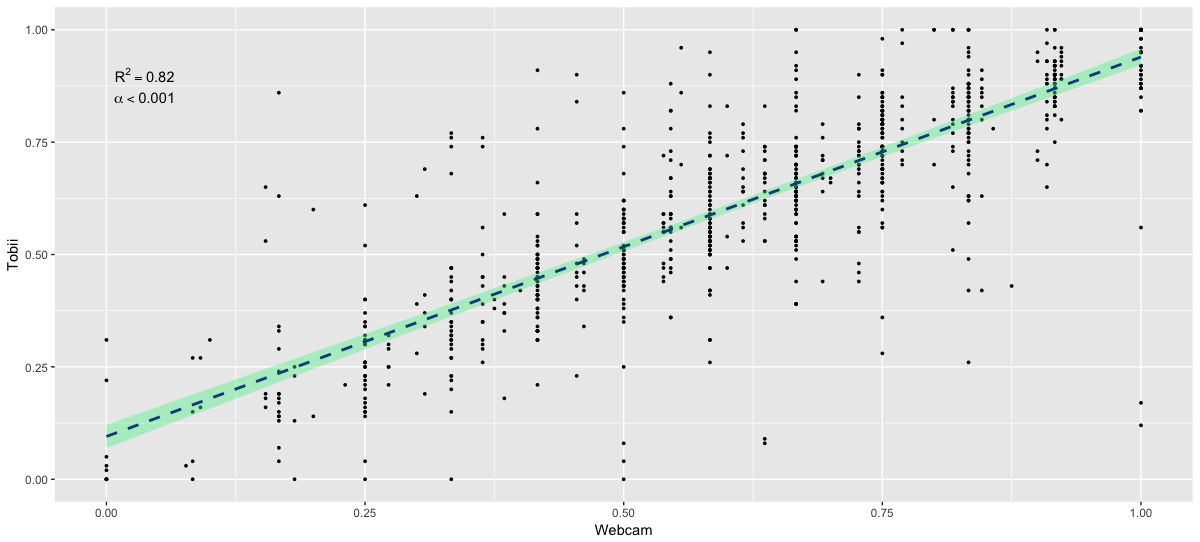
Relationship between trial-level novelty preference scores collected by the Tobii commercial-grade eye tracker and device-embedded web camera.

Next, we compared the relationship between data from the commercial grade eye tracker camera and the laptop-embedded camera for each of the 20 test trials per participant. Analyses revealed robust associations between each camera type across test trials, with a Spearman’s Rho correlation of .82 (n=841, *P*<.001; Figure 2). Inter-rater reliability of scoring the laptop embedded camera data using Krippendorff’s kappa formula revealed a strong agreement between the three human raters for each of the 15 frames across each of the 20 test trials (kappa=.84).

## Discussion

The primary focus of this study was to examine the relationships between VPC performance and traditional cognitive assessments known to be sensitive to signs of early cognitive dysfunction. Previous studies have shown differential performance on the 30-minute VPC task between various cognitive subgroups [11], as well as the predictive value of the task in identifying individuals who will progress from normal cognitive function to amnestic MCI (aMCI) or from aMCI to AD within three years of the assessment [12]. However, these previous studies only collected data with commercial-grade eye trackers and did not investigate the correlation between VPC performance and cognitive composite scores. Our results demonstrate convergent validity between a 30-minute VPC eye-tracking task and both the PACC and NIHTB-CB batteries. This investigation presents the first data demonstrating modest to moderate correlations between VPC task performance using device-embedded cameras and scores on gold standard cognitive composites, suggesting these eye-tracking-based tests can provide complementary support to conventional cognitive composites for detecting early cognitive changes.

We also discovered that the observed correlations between VPC performance and cognitive battery scores were driven by particular cognitive domains. Specifically, VPC performance correlated the highest with measures of processing speed, executive function, and visual episodic memory. While a trend association was seen on a measure of verbal episodic memory, the exclusively visual nature of the VPC task would be expected to drive a stronger relationship with other measures of visual episodic memory. VPC task performance has previously been shown to be associated with processing speed [30], which accounts for a large proportion of variance across cognitive tasks, including executive functioning [31].

The last major focus of this study was to explore the relationship between VPC performance data collected by distinct camera types. To our knowledge, this is the first study to measure the correlations between VPC task data collected from both commercial-grade and device-embedded cameras for a 30-minute VPC task. Commercial-grade eye-tracking technologies have been shown to detect abnormal eye movements across a number of clinical populations, including people with schizophrenia [32], autism [33], ADHD [34], multiple sclerosis [35], and cognitive decline [36]. These high frame rate cameras typically collect an abundance of data on eye movement behavior, including saccades, gaze fixation and duration, smooth pursuit, and other metrics that can provide valuable insights for certain cognitive processes [19,20]. However, these devices are used primarily in research settings due to their complexity and high cost, so the need exists for an alternative eye-tracking system that is feasible for more widespread use. This study demonstrates that data collected from webcams at 30 FPS that is subsequently down-sampled to 3 FPS can provide clinically relevant insights into cognitive function. As such, the robust datasets collected by commercial-grade (ie, 60 FPS) cameras are not always necessary for certain assessments, such as VPC tasks.

Research on methods for the recording and analyzing eye movements from device-embedded web cameras continues to grow [37-44], demonstrating the utility of real-time online systems and offline recording systems. Perhaps the most significant advantage of built-in web cameras is their lack of geographical restriction to collect eye feature data on large samples sizes. For example, open source eye-tracking software, such as WebGazer.js [45] can be deployed across most major web browsers to provide insight into the eye movements of website visitors. The widespread reach of device-embedded cameras has the potential to greatly increase access to eye-tracking-based cognitive assessments across geographically dispersed populations, as people can take the tests anytime in their own homes.

These results further demonstrate that both commercial-grade eye trackers and device-embedded cameras can produce robust data of sufficient quality for analyzing VPC task performance. High correlations existed between VPC performance using commercial-grade devices and device-embedded cameras at both the overall and trial level, suggesting that webcams represent a consistent, scalable, and reliable method for VPC data collection. These findings align with results from a previous study, in which we demonstrated strong associations between manually scored data from a device-embedded camera and automatically scored data from a commercial-grade eye tracker for an abbreviated 5-minute version of the VPC task [21]. The growing evidence base supporting the comparability of VPC data between commercial-grade and embedded cameras is an important development for the field of remote cognitive assessments.

The scalability and lower cost of the webcam-based VPC task holds the potential to greatly increase screening rates for early signs of cognitive decline, which will be an important component of caring for ever-growing aging populations worldwide in the coming decades. While the gold standard PACC and NIHTB-CB cognitive assessments are reliable for detecting preclinical cognitive decline, they are also limited in their scalability, much like commercial-grade eye-tracking devices. The cognitive composites must be taken in person, to ensure standardized administration by a trained professional who can guide participants through the various sets of instructions. Conversely, the webcam-enabled VPC task is better suited for widespread adoption because it is reliable, language-agnostic, requires little to no instruction and minimal equipment, and can be administered and completed anywhere. The VPC task using eye-tracking data collected from web cameras is a potential complement to traditional test batteries for cognitive decline. The further integration and development of these scalable tasks by companies like Neurotrack Technologies, Inc, (Redwood City, CA) will greatly increase the availability of these assessments.

This study has a few limitations. For one, the small study sample comprised of clinically normal older adults restricts the generalizability of the results to broader populations. However, we were able to recruit a diverse group of participants and will strive to do so in larger studies in the future to maximize the external validity of the results. Also, the collection of the webcam-based VPC data within a clinic setting is not ideal for approximating in-home performance, but the validation of webcam-based data in a research setting is a necessary precursor to remote data collection.

These results set the stage for many future directions. We demonstrated here that manual scoring of the webcam-based VPC task had high inter-rater reliability, indicating that this method of data quantification produces consistent results across different scorers. The future development of an automated scoring system for device-embedded camera data would be extremely valuable, allowing for faster scoring and deployment on a larger scale. Additionally, although outside of the scope of this study, future studies will need to examine the test-retest reliability of the webcam-based VPC tests to ensure high internal validity.

In conclusion, this study showed strong convergence in data accuracy between commercial-grade eye tracking cameras and device-embedded cameras on a 30-min VPC task. Results demonstrated modest to moderate correlations on 30-minute VPC task performance using device-embedded cameras and performance on gold standard digital and paper-pencil cognitive composites. Eye tracking through device-embedded cameras can provide efficient and scalable evaluation of cognitive performance and support the growing number of individuals at risk for cognitive decline.

## Abbreviations

AD: Alzheimer disease
aMCI: amnestic mild cognitive impairment
DCCS: Dimensional Change Card Sort Test
FLV: flash video
FPS: frames per second
MCI: mild cognitive impairment
NIHTB-CB: National Institutes of Health Toolbox Cognitive Battery
NP: novelty preference
PACC: Preclinical Alzheimer’s Cognitive Composite
PCPST: Pattern Comparison Processing Speed Test
PSMT: Picture Sequence Memory Test
PVT: Picture Vocabulary Test
VPC: visual paired-comparison
